# A 4D printed self-assembling PEGDA microscaffold fabricated by digital light processing for arthroscopic articular cartilage tissue engineering

**DOI:** 10.1007/s40964-022-00360-0

**Published:** 2022-11-09

**Authors:** Yunjie Hao, Chuanyung Wu, Yuchuan Su, Jude Curran, James R. Henstock, Fangang Tseng

**Affiliations:** 1 Department of Engineering and System Science, National Tsing Hua University, Hsinchu, 30013 Taiwan; 2 Department of Engineering and System Science, Frontier Research Centre On Fundamental and Applied Sciences of Matters, National Tsing Hua University, Hsinchu, 30013 Taiwan; 3 Research Centre for Applied Sciences, Academia Sinica, No. 128, Sec. 2, Academia Rd., Nankang, 11529 Taipei Taiwan; 4 Department of Mechanical, Materials and Aerospace, School of Engineering, Harrison Hughes Building, University of Liverpool, Liverpool, L69 3GH U.K.; 5 Institute of Life Course & Medical Sciences, William Henry Duncan Building, University of Liverpool, Liverpool, L7 8TX U.K.

**Keywords:** DLP-3D printing, PEGDA hydrogel, Articular cartilage, Tissue engineering, Self-assembly scaffold

## Abstract

Articular cartilage in synovial joints such as the knee has limited capability to regenerate independently, and most clinical options for focal cartilage repair merely delay total joint replacement. Tissue engineering presents a repair strategy in which an injectable cell-laden scaffold material is used to reconstruct the joint in situ through mechanical stabilisation and cell-mediated regeneration. In this study, we designed and 3D-printed millimetre-scale micro-patterned PEGDA biomaterial microscaffolds which self-assemble through tessellation at a scale relevant for applications in osteochondral cartilage reconstruction. Using simulated chondral lesions in an in vitro model, a series of scaffold designs and viscous delivery solutions were assessed. Hexagonal microscaffolds (750 μm x 300 μm) demonstrated the best coverage of a model cartilage lesion (at 73.3%) when injected with a 1% methyl cellulose solution. When chondrocytes were introduced to the biomaterial via a collagen hydrogel, they successfully engrafted with the printed microscaffolds and survived for at least 14 days in vitro, showing the feasibility of reconstructing stratified cartilaginous tissue using this strategy. Our study demonstrates a promising application of this 4D-printed injectable technique for future clinical applications in osteochondral tissue engineering.

**Supplementary Information:**

The online version contains supplementary material available at 10.1007/s40964-022-00360-0.

## Introduction

Articular hyaline cartilage is a specialised connective tissue which covers the interfacing ends of hard bones and forms synovial joints such as the knee [1]. As a biological shock absorber, cartilage is easily damaged by high impacts, excessive wear and disease, but damage is difficult to diagnose at very early time points, and adult cartilage has a poor innate ability to self-repair [2]. Clinical options for repairing small defects in joint cartilage depend on the size, site, and severity of the lesion and may include both non-operative pharmaceutical approaches and minimally invasive arthroscopic surgery. These solutions are usually merely palliative, emphasising pain relief over actual reconstruction of the tissue, and ultimately only delay the eventual requirement for a prosthetic joint replacement [3]. Although the ongoing lack of an effective gold standard in truly regenerative cartilage surgery, tissue engineering (TE) offers potential for recreating functional anatomical structure and enabling return to normal physiological function [4]. As a result of the high clinical incidence of cartilage traumas and low-cost of current therapies, strategies in which tissue engineered substitutes can be pre-designed and mass manufactured to act as ‘off the shelf’ tissue grafts are of substantial interest [5, 6].

The most common clinical presentation of early-stage cartilage defects is irregular, small lesions requiring arthroscopic (keyhole) repair, and so injectable strategies bring the most favourable regenerative medicine approach for pre-osteoarthritic joint reconstruction [7]. Joint arthroscopy is commonly performed as a minimally invasive surgery to remove cartilage debris and debride the edges of lesions, and so the simple administration of a restorative hydrogel as a follow-up procedure holds promise in being an attractive option for clinicians [8, 9]. Whilst traditional solid scaffolds for cartilage regeneration are pre-shaped before application, injectable scaffolds can be formed or self-assembled in situ to completely fill a defect [7, 10, 11]. One disadvantage of this approach is the mismatch of mechanical properties between the injected biomaterial (which is often a type of hydrogel, e.g. collagen or polyethylene glycol-based) and the native cartilage, which has a complex gradation of varying compressive and tensile properties between the deep zone and superficial layers [12–14]. Whilst the stiffness of injected hydrogels can be improved by the inclusion of composites materials (e.g. crystalline, high aspect ratio fibres such as nanocellulose [15], this mismatch is associated with poor clinical outcomes, since the joint does not resume functional strength and body weight loads on the defect can cause further wear or damage to the repair matrix [12]. With this in mind, our approach has been to generate a composite injectable biomaterial which contains a self-assembling porous microscaffold which acts to reinforce the mechanical integrity of the repaired lesion.

The biocompatibility of the scaffold used for regenerating cartilage tissues is critical to the function of the engineered tissue construct and post-operative success. Incorporating an appropriate biomimetic 3D architecture into the scaffold has been shown to support the proliferation of cells, either those naturally resident in the joint or supplemental through a combinational cell-material delivery strategy. Considerable research has accumulated relating to 3D architectural parameters such as porosity, surface chemistry and mechanical properties which influence the recruitment, attachment, proliferation, migration and differentiation of reparative cells [16, 17]. In essence, the requirement for these biomaterial scaffolds is that they closely match the physical, mechanical and chemical properties of the native tissue, and whilst synthetic hydrogels fulfil many of these requirements, a more structured porosity at the cellular scale is required to fully optimise their biomimetic properties and support effective cell engraftment [18]. An array of injectable scaffolds has been developed, including biopolymer hydrogels such as collagen, semi-solids and gelled or polymerised solids such as polymethylmethacrylate cements, porous cryogels, micro/nano-particles, microspheres, nano-films, bio-ceramics, foams and combinations of these materials [19–22].

In this study, an injectable self-assembling PEGDA-based microscaffold was designed for the purpose of reconstructing the deep zone of articular cartilage tissue. The microscaffold was designed and fabricated using a customised Digital Light Processing (DLP) 3D printer, and our specific requirement of generating 4D in situ self-assembling macrostructures were investigated. The design was optimised to match the mechanical properties of deep-zone articular cartilage, and standard tissue engineering considerations of pore size and interconnectivity were embedded in the design criteria to ensure effective cell migration, diffusion and nutrient/waste mass transport [23–25]. High resolution 3D printing using DLP provides highly flexible control over the generation of various materials, with rapid processing, inexpensive fabrication, and the ability to manufacture complex micro-patterned structures using photo-crosslinked hydrogels [26, 27]. Self-assembly process at micro- and nano-scales can be driven by geometric patterns, molecular recognitions, chemical or electrostatic interactions, and tailored to arise automatically or in response to external stimuli or under specific conditions [28–30], with applications including cartilage tissue fabrication [31–33].

3D printing is developing as an essential component of modern biomedical engineering, having already found applications in personalised internal prosthetics [34], in situ bioprinted tissue grafts for cartilage repair [35], and as an open design tool for innovation in tissue engineering [36]. 4D printing is an exciting revolution in additive manufacturing, in which the 3D printed material subsequently undergoes a predetermined morphological change (e.g. directed self-assembly) in response to an external stimulus [37]. The extensive potential for novel applications of 4D printing in tissue engineering and medicine is now emerging, including self-assembling and self-healing biocompatible polymers, and materials that can respond to externally applied stimuli such as heat, light or magnetic fields [38, 39].

Our objective in this investigation was to develop a 4D approach in which the individual 3D printed microscaffold components self-assemble after arthroscopic injection into a final structure which is stabilised by both the designed microscaffold geometry and by the action of embedded cells in response to the environmental conditions. Our hypothesis was that the addition of live, reactive cells into micropores within the material would enable the 3D printed microscaffolds to facilitate improved healing over time in response to the wound microenvironment, including the biochemical signals from surrounding tissue, and in response to the mechanical requirements as the joint returns to function through extracellular matrix organisation [40].

## Materials and methods

### 3D printing of biomaterials

**Fig. 1:**
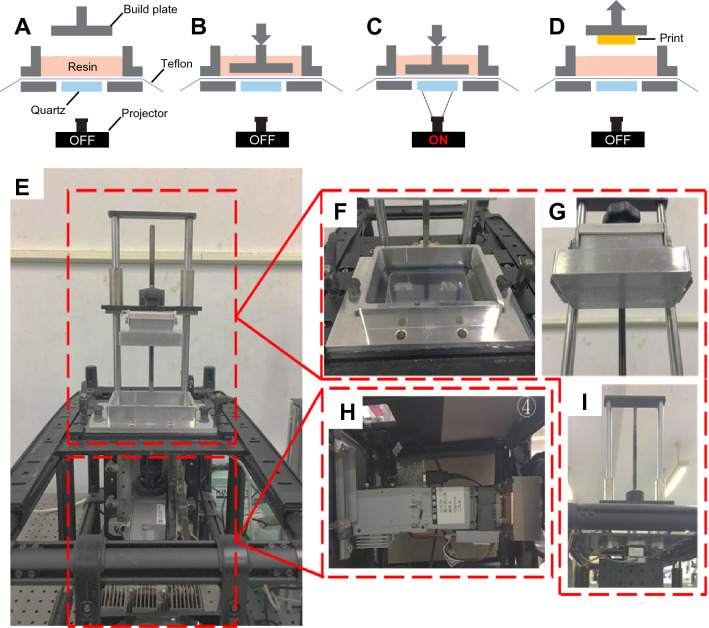
The customised digital light processing (DLP) 3D printer and 3D printing process. Schematic images of the printing process for the customised DLP 3D printer (**A**-**D**) show the four stages involved in microscaffold fabrication: **A** Preparation, **B** Lowering of the build platform into the resin solution, **C** Photoinitiation, and **D** Removal of the printed material. Image showing the configuration of the customised DLP 3D printer (**E**), including resin bath (**F**), build platform (**G**), the projector containing (UV) light source and DMD array (**H**) and the motor engine (**I**)

Biomaterial scaffolds were fabricated using a customised Digital Light Processing (DLP) stereolithography 3D printer (Fig. 1). Our customizable 3D printer was designed to enable more direct control over the printing process, and was modular to enable versatile development of our workflow. We built a customised 3D printer using the components typically found in commercially available DLP printers, with modularized and upgraded lighting and projection subsystems to realise image output with multiple/switchable wavelengths and high resolutions. Meanwhile, we utilised an open-source software approach and G-code (i.e. the most widely used computer numerical control programming language) to achieve direct and precise hardware control over the printing process.

A high-resolution UV projector (NVM UV series, Young Optics) with a 365 nm LED, and a DLP4710 digital 1920 × 1080 array micro-mirror device (Texas Instruments) was used to project images for layer-by-layer polymerisation. To achieve the desired control throughout the polymerisation process, both light intensity (in 1024 levels with a maximum output of 500mW) and exposure time (in milliseconds) were adjusted independently, with the standard protocol for a single layer (10 μm) of resolution test models being 21.1 mW/cm^2^ light intensity delivered over 2000 ms. The final protocol for generating cell-laden microscaffolds was established using 50 μm layer thickness exposed to 5.859A light for 4000 ms (35.2 mW/cm^2^). The microscaffold size was 1–1.2 mm in diameter, with 500-600 μm thickness, with 100–200 μm diameter hole patterns. The pitch of the micro-mirror array was 5.4 µm, resulting in an output image resolution of 18 µm when using a 1:3.33 projection lens. A Solus 3D printing platform (Reify Technology) and an open-source Creation Workshop program were utilised to control and synchronise the printing process. The Z resolution (i.e. the minimum layer thickness) of the 3D printing platform was 5 µm, and the maximum build volume was 34.56 × 19.44 × 50 mm [41]. All mask patterns were pre-designed in Solidworks®, transferred to the computer connecting to the printer and further edited by Creation workshop (CW) software to generate G-code.

### Biomaterial

Poly (ethylene glycol) diacrylate (PEGDA Mn 700, Sigma) was used to construct the biomaterial microscaffold with custom designed patterns. Biomaterial inks were composed of 20–30% PEGDA with 0.05% lithium phenyl-2,4,6-trimethylbenzoylphosphinate (LAP) as the photoinitiator, additional 0.05% tartrazine as a light absorber in printing test models, and 0.05–0.2% nanocellulose fibres (Echo Chemical Co. Ltd, Taiwan) in preliminary experiments as described below. All reagents were dissolved in water with no organic solvents to maximise biocompatibility. The PEGDA-based ink was mixed thoroughly and poured into a vat with a transparent Teflon film base, supported by a transparent quartz plate.

### Mechanical testing and resolution

Preliminary experiments were conducted to examine the compressive moduli, weight degree of swelling and kinetic dehydration profile of either 20% or 30% PEGDA biomaterials with/without additional nanocellulose fibres (NCF) (0%, 0.005%, 0.02%, 0.2%) over 8 h. Biomaterials were printed in a 10 × 10 × 6 mm block for unconfined uniaxial compressive testing (ElectroForce 3100, TA instrument). To optimise the resolution regarding the aspect ratio and shape fidelity for the final microscaffold design, test models of convex/concave patterns in a series of diameters (30, 50, 100, 200, 300, 400, 500 µm) were evaluated. The hexagonal microscaffolds were firstly designed to be 1.0–1.2 mm in diameter of the inscribed circle of the hexagon, 500–600 µm in thickness, with 100-200 µm patterning of holes (diameter and spacing). The quality of printed microscaffolds was checked using an inverted microscope after printing and kept in distilled water in centrifuge tubes at 4 °C prior to use.

### Self-assembly and infill of a simulated cartilage lesion

The self-assembly property of the microscaffold was characterised by analysing the coverage rate after injecting the microscaffold suspension into a hole which was used to simulate the small chondral lesion in the femoral condyle. Microscaffolds were collected in the base of the centrifuge tube by gently centrifuging, and the supernatant water was replaced with a viscous delivery solution. After filling the syringe, the microscaffold-suspended solution was injected into the 3D-printed resin with a small hole (1 cm in diameter and 2 mm in thickness). The flow rate and velocity of injection were controlled by manually extruding the solution at 1 ml/minute. Images of the area covered by microscaffolds were recorded by a camera to quantify the self-assembly property of the patterned scaffold after injection. Every test of individual sample was repeated 10 times for statistical analysis. Images were processed using Image J (v1.8.0.112, USA) [42] into 8-bit black and white images allowing covered versus uncovered areas to be discerned and quantified as pixels.

To optimise the biomaterial, a series of microscaffolds with uniform hole patterns (100-200 µm) but varied diameters (0.75, 1.05, 1.35, and 1.5 mm) and thicknesses (0.3, 0.4, 0.55 and 0.6 mm) were developed. To optimise the process of injection, solutions with various viscosities were prepared to suspend microscaffolds for evaluating the self-assembly property. These included pure water with viscosity of 0.89cp and methyl-cellulose (M7140, Sigma) solutions at three different concentrations (1%, 2% and 4%), yielding solutions with approximate viscosities of 7.5cp, 15cp and 30cp respectively.

### Cell culture

To evaluate the morphology of cells growth directly on the surface of PEGDA scaffolds, ATDC5 cells (Chondrogenic cell line, European Collection of Authenticated Cell Cultures) were seeded and cultured on PEGDA-based scaffolds with/without collagen coating (0.1 mg/ml porcine collagen type I solution, SusGel™, Conori Inc.). For cell morphology observations on 3D culture, cells were suspended in type I collagen gel (2.7 mg/ml, SusGel™) at a concentration of 105 cells/ml. The collagen gel was used as a viscous carrier to enable mixing of cells with the 3D biomaterial microscaffolds and facilitate engraftment. Microscaffolds with different patterns were printed and soaked into pure water for up to three days to leach out potential toxicants, then sterilised by immersion in 75% alcohol followed by UV light exposure in a biosafety cabinet for 2 h. A subset of microscaffolds were additionally coated with collagen (0.1 mg/ml porcine collagen type I solution, SusGel™, Conori Inc.) by immersing them into the solution, draining the excess to leave a thin coating, and subsequent drying at room temperature for 1–2 h. Both coated and non-coated microscaffolds were transferred into 24-well microplates, and put in an incubator after cell seeding. Culture medium was replaced every two to three days.

### LAP cytotoxicity

Cytotoxicity of the photoinitiator (LAP) was determined by lactate dehydrogenase (LDH) assay, using three cell lines: NIH 3t3 fibroblasts (American Type Culture Collection, ATCC), ATDC5 (European Collection of Authenticated Cell Cultures, ECACC) and C2C12 myoblasts (ATCC) in 24-well microplates. The seeding cell densities were set at 5 K, 10 K, 20 K, 40 K and 80 K cells per well for NIH3t3, and 10 K, 20 K, 50 K, 100 K and 500 K cells per well for both C2C12 and ATDC5. Cells were cultured and maintained with growth medium (DMEM or DMEM/F12 (Corning®) supplemented with 10% foetal bovine serum (HyClone™, GE healthcare Life Sciences) and 1% penicillin/streptomycin (Corning®)) in T75 plastic tissue culture flasks (Thermo Scientific™) in an incubator at 37℃ with 5% CO2. Three concentrations of LAP (0.05%, 0.1% and 0.15%) were made in growth medium from a 1% stock solution (dissolved in pure water (Merck Millipore Milli-Q™) and sterile filtered with a 0.22 µm filter (Sartorius)), and added to cells pre-seeded in 24-well microplates. The culture medium of each well was replaced every two or three days, and LDH assay was performed on the media after 1, 4, and 7-day treatments. Growth medium containing 5% DMSO was used as a positive control group for cytotoxicity. LDH assay was performed according to the manufacturer’s instructions for the CyQUANT™ LDH Cytotoxicity Assay (Invitrogen™), with assay detection at 490 nm and reference wavelength at 680 nm. All results about the cytotoxicity were shown as the cell viability in percentage.

### Biocompatibility testing

The biocompatibility of 3D-printed PEGDA was determined by MTT assay in accordance with the ISO standard (ISO10993-12:2021). After printing, bulk PEGDA hydrogels (10 mm × 10 mm × 0.8 mm) were soaked in DMEM (1 ml per sample) and incubated at 37 °C for 72 h to collect the leachate from freshly printed PEGDA. To determine the effectiveness of aqueous washing to remove toxic species from PEGDA scaffolds, additional samples were pre-soaked for 72 h in 50 ml deionised water prior to the 72 h DMEM incubation process. Supernatants of the fresh and pre-washed PEGDA-incubated solutions were collected and filtered (0.22 µm) to sterilise. DMEM was used as a negative control, with DMSO as a positive control. To screen the MTT cell viability/cytotoxicity, NIH 3T3, C2C12 and ATDC5 cells were separately seeded into 24-well microplates and cultured in an incubator for 24 h. The culture medium was then replaced with the conditioned test medium and assayed after 1, 4 and 7-days incubation. To perform the MTT assay, culture medium was replaced by 1 mg/mL MTT (Thiazolyl Blue Tetrazolium Bromide, Sigma) solution in growth medium and incubated for 4 h. The supernatant of each well was gently removed and discarded, and replaced by acidic isopropanol solution (aIPA) [43]. The plate was gently shaken to dissolve the purple formazan salt into isopropanol, then samples of these solutions from each group were transferred into a 96-well microplate to quantify the absorbance of each well at 570 nm wavelength by an automated plate reader (BioTek™, Agilent Technologies, Inc., USA).

A Live/Dead assay was used to examine the viability of cells cultured with the 3D-printed PEGDA scaffolds. The staining process was performed according to the instruction from the manufacturer (LIVE/DEAD Viability/Cytotoxicity Kit, for mammalian cells (ThermoFisher, L3224). In brief, samples were taken out of the incubator and checked under normal inverted microscope. Culture medium was replaced with staining solution containing green-fluorescent calcein-AM (2 µM working solution) and red-fluorescent ethidium homodimer-1 (4 µM working solution) diluted in growth medium. Samples were incubated for 40 min and then carefully washed twice with PBS before being transferred gently onto the microscope slides using a sterilised tweezer for further confocal microscope examination.

Cells were stained for cytoskeletal actin to determine cell engraftment within the biomaterial microscaffolds. After 7–14 days culture samples were carefully washed with PBS and fixed in 4% paraformaldehyde (Sigma) solution at room temperature for 1 h. After rinsing with pure water, a staining solution containing the high-affinity fluorescence-conjugated antibody for cytoskeletal F-actin (AlexaFluor™488 phalloidin, Thermo Scientific™) was added. Samples were incubated at room temperature for 30 min in dark and washed twice in PBS before examination with a fluorescence microscope (Olympus IX70 or Zeiss LSM 800).

### Statistical analysis

Numerical data from MTT and LDH assays, mechanical properties, swelling ratio, and coverage rate were statistically analysed by Microsoft® Excel and Origin2018 (OriginLab®, USA). The differences between groups were calculated by ANOVA one-way test (Tukey post hoc test), and linear regression analysis was performed on cell culture numbers. A p value of 0.05 was considered the threshold for statistical significance.

## Results and discussion

**Fig. 2:**
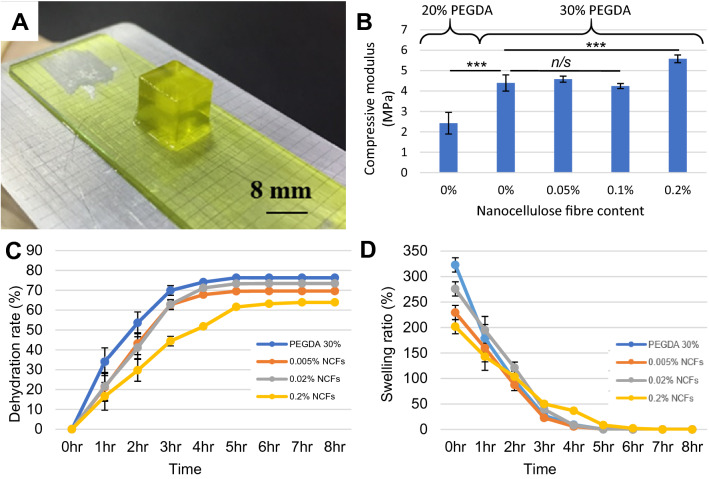
Alteration of PEGDA scaffold physical and mechanical properties using nanocellulose fibres. 8 mm cubes of either 20% or 30% PEGDA and 0–0.2% nanocellulose fibres were produced for physical testing (**A**). Compressive modulus was analysed (**B**), and the dehydration (**C**) and swelling (**D**) properties compared using formulations of varying nanocellulose incorporation. The scale bar (A) is 8 mm. Statistical analysis was performed using a one-way ANOVA followed by at Tukey’s post hoc test, error bars show standard deviation (*n* = 9), (*n/s*—data not statistically significantly different, ***p < 0.001)

A 3D printing apparatus was successfully developed using commercially available components, consisting of a movable build plate, resin vat with quartz window, and a DLP projector using digital mask (Fig. 1). The DLP printing procedure is based on a peel/squash method using a fluorinated ethylene propylene (FEP) film to reduce adhesion, and has a modularized design with upgraded light source and projector subsystems to realise image output with multiple/switchable wavelengths and high resolutions using open-source software and g-code to achieve precise control over the printing process. The composition of PEGDA used to generate microscaffolds was developed by comparing the relative mechanical properties of 20% versus 30% PEGDA, and 0–0.2% nanocellulose fibres (Fig. 2). The compressive modulus of 30% PEGDA was shown to be significantly greater than 20% PEGDA (1.9-fold increase), and at 4.4 MPa was comparable to the compressive modulus of deep-zone cartilage, which was the target tissue for engineering this repair strategy [24, 25]. The addition of 0.2% nanocellulose fibres resulted in a further increase in compressive modulus, from 4.4 MPa (without fibres) to 5.6 MPa (with 0.2% fibre inclusion), although lower levels of inclusion did not result in a detectable increase in strength (Fig. 2B). The dehydration rate of printed 30% PEGDA with 0.2% NCF shown to be evidently slower than either PEGDA alone or with lower levels of nanocellulose inclusion (Fig. 2C), but swelling ratio was unaffected by incorporation of nanocellulose in the gel matrix (Fig. 2D).

**Fig. 3:**
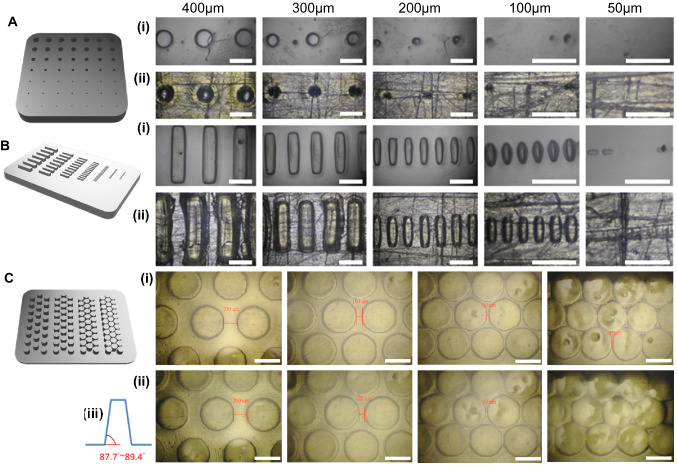
Optimization of 3D printing parameters. Resolution tests (400-50 μm) were performed at 1 mm thickness using either a concave circular mask (**A**) or convex cuboid mask with 4:1:1 geometry (**B**), using PEGDA alone (Ai and Bi) or with tartrazine as an additional light absorber (Aii and Bii). Shape fidelity studies were performed using PEGDA plus 0.05% LAP and 0.05% tartrazine light absorber with a convex mask with a 2:5 aspect ratio, and printed at 10 μm per layer with a 2 s per layer exposure time for a total of 50 layers (**C**). A convex rod mask with identical rod geometries but a series of interval spaces of 300, 200, 100 and 50 μm was used to determine shape fidelity between the top layer (Ci) and bottom layer (Cii) of the resulting printed patterns. Fidelity of interval spacing between the top and bottom layers was maintained at 300 μm (270-260 μm), 200 μm (120-160 μm), and 100 μm (30-60 μm) but lost at 50 μm. The images enabled the angle of inclination of the convex pattern to be calculated as 87.7–89.4°. Scale bar is 500 μm

**Fig. 4:**
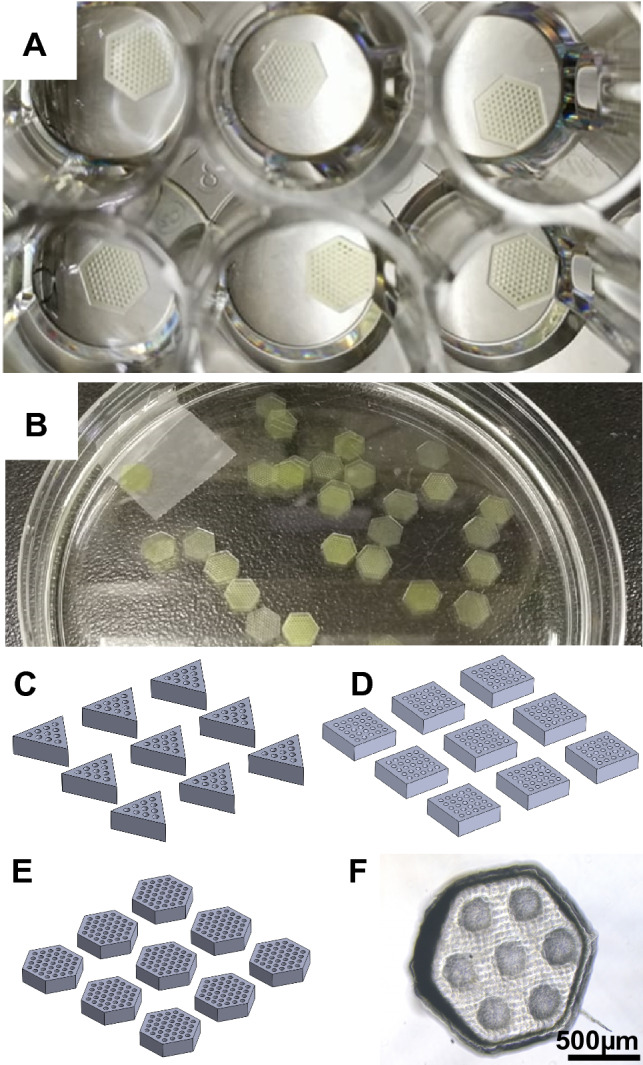
3D-printed PEGDA self-assembling. microscaffolds. Self-assembling scaffolds were printed and stored in deionized water in wells of 24-well microplates (**A**) and petri dishes (**B**) for cell culture. Schematic images of different designs of the self-assembly hydrogel scaffold, with triangular (**C**), square (**D**) or hexagonal (**E**) contour profiles and uniform hole arrays (100–200 μm in diameter and separation). The miniaturised self-assembling PEGDA microscaffold printed with the final, optimised parameters (**F**). Scale bar in F is 500 μm

The 3D printing resolution and shape fidelity was established by a series of studies (Fig. 3). Concave patterns printed in the layer-by-layer mode with additional light absorber (0.05% tartrazine) were shown to generate improved resolution compared to patterns printed in single-layer printing mode. Convex patterns either with or without light absorber and using both printing modes yielded similar print resolutions with no discernible differences. Under comparable printing conditions, convex patterns presented better resolution in our DLP-3D printer. We theorise that this results from the incident light energy dispersion phenomenon which could not be entirely eliminated by adding a light absorber to control the light energy distribution during printing. The aspect ratios achievable by our DLP printer were 1:5 for convex patterns and 3:5 for concave patterns, resulting in a small angle (~ 2°) for printing vertical structures due to light scattering and superposition of the exposure. The degree of this angle was dependent on the distance between neighbouring convex patterns. Following these studies, a final printing ink of 30% PEGDA with 0.05% LAP was determined as a suitable formulation for ongoing studies into microscaffold geometries with sufficient capacity for generating geometric patterns at the required level of resolution, reproducibility and production yield (Fig. 4).

**Fig. 5:**
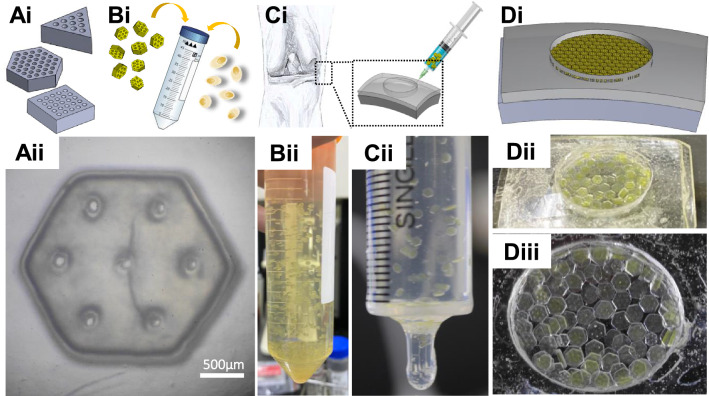
Characterisation of the PEGDA-based microscaffold self-assembly properties. **Ai**: Schematic images of three geometric designs for the self-assembly microscaffold: triangular, hexagonal, and square outer profiles with uniform hole arrays (100–200 μm in diameter and spacing), and the final microscaffold design used for subsequent studies (**Aii**). **Bi**: To perform the self-assembly in injectable solutions, PEGDA scaffolds were suspended along with cells in liquids with different viscosity, forming a homogenously dispersed suspension (**Bii**). Ci: The suspended scaffolds were injected into an artificial 1 cm diameter cartilage lesion via a syringe to mimic an arthroscopic repair procedure (**Cii**), ultimately simulating a full-thickness ‘repair’ (**Di** and **Dii**). The coverage rate of scaffolds was measured by analysing photographic images taken from underneath the transparent defect mould (**Diii**). The scale bar in Aii is 500 μm

To assess the ability of the microscaffolds to fill a macroscopic cartilage lesion, an idealised model simulating a small chondral defect with 1 cm in diameter and 2 mm deep was 3D-printed using a colour clear printing resin the NextDent Ortho Clear (Vertex-Dental B.V., Soesterberg, The Netherlands) (Fig. 5). Assisted by the model, an arthroscopic procedure could be performed, where autologous cells are mixed with a biocompatible collagen hydrogel, and the ‘off the shelf’ sterile microscaffolds providing the mechanical strengthening and a biological scaffold to better effect cell ingrowth and healing. The procedure was briefly optimised to determine that visualisation by photographic imaging of the underside of the defect model, followed by digital processing of the images into black and white pixelated data files enabled the overall coverage rate to be quantified.

**Fig. 6:**
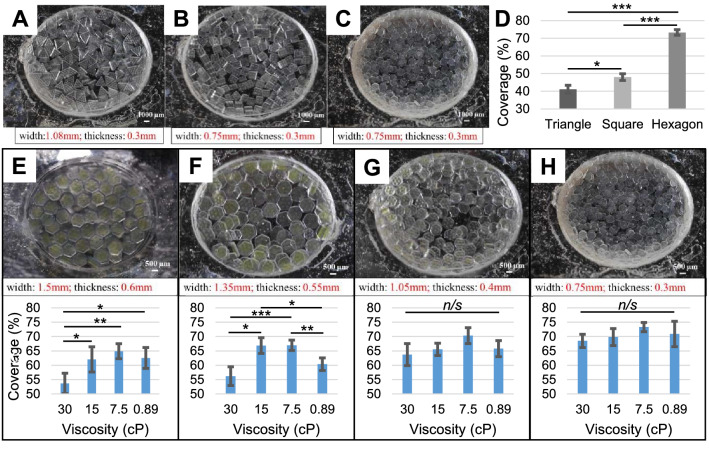
Coverage rate analysis of self-assembling scaffolds with varying sizes, contour profiles and delivery solution viscosity. Self-assembling microscaffolds with were printed with similar sizes but varied contour profiles: **A** Triangular, **B** Square, and **C** Hexagonal. The coverage ability of each geometry was compared following injection into the model defect in a 7.5cp viscosity hydrogel solution (**D**). Four scales of the patterned self-assembling scaffolds were printed by varying the diameter and thickness, and their coverage rates compared after injecting them into the defect model in suspensions with four different viscosities (**E–H**). The scale bar is 1000 μm in A-C and 500 μm in E–H. Statistical analysis was performed using a one-way ANOVA followed by at Tukey’s post hoc test, error bars show standard deviation (*N* = 10), (*n/s* – data not statistically significantly different, *p < 0.05, **p < 0.01, ***p < 0.001)

The ability of the microscaffolds to tesselate into a continuous layered structure with semi-regular architecture (self-assembly properties) was then assessed by analysing the coverage rate after injection (Fig. 6). In initial experiments, microscaffolds with similar sizes but different exterior shape profiles were assessed (Fig. 6A–D). A solution with 7.5cp viscosity was used to suspend triangular, square and hexagonal microscaffolds to compare relative coverage rates. Hexagonal microscaffolds showed the best coverage rate (73.3%), confirming our hypothesis that the innate ability of hexagonal shapes to self-organise into contiguous macrostructures. Coverage was not perfect, however, with occasional gaps in the centre of the model, and overlapping at the edge which we postulated to be caused by uneven extrusion force and suboptimal rheological properties of the viscous microscaffold colloidal suspension, and forms the basis for our ongoing studies. The correlation between hexagonal shape and self-assembly feature has been observed at multiple scales in a wide variety of instances, such as nanoparticle aggregates, nanocrystal superstructures, micro/nano-scaled molecular sequence motifs, and mesoscale patterns [44–50]. Our data support the concept that this self-assembling property extends to millimetre scales and can be exploited for biomaterials applications in 4D printing and tissue engineering. We propose that the microinjected scaffold assembly will stabilise in situ through tessellation of the microscaffold shapes, further crosslinking of the collagen in response to environmental conditions, and through the action of the cells to generate and remodel new extracellular matrix. Additional stabilisation and crosslinking strategies to contain the reconstructed material would likely be required in clinical translation, e.g. superficial suturing of materials such as Chondro-Gide over the implant to prevent loss of microscaffolds, rest or external joint stabilisation.

To further test the effect of the microscaffold size and viscosity of the suspension hydrogel on coverage rate, hexagonal microscaffolds with four different scales were prepared and suspended in solutions with four different viscosities ranging from 30 to 0.89 cP (Fig. 6E–H). Microscaffolds with the smallest size (0.75 mm in width and 0.3 mm in thickness) demonstrated the highest coverage rates in each suspension solution (68.5—73.3% coverage). A solution with 7.5 cp viscosity was shown to give the highest coverage rate with all microscaffold geometries, and this was standardised for subsequent experiments.

**Fig. 7:**
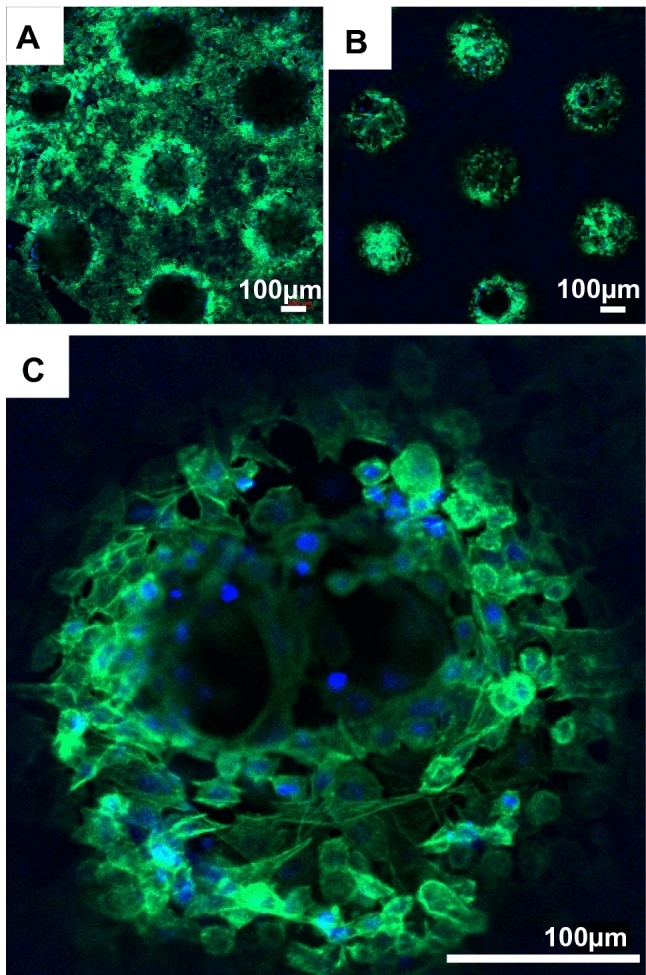
Cells cultured on 3D-printed self-assembling PEGDA microscaffolds. ATDC5 chondrocytes were seeded on 3D-printedscaffolds in a collagen hydrogel, and cultured in vitro for 14 days prior to staining for cytoskeletal F-actin (green) and the nuclear DAPI staining (blue). Confocal microscopy images show the morphologies of cells engrafted on the surface of the scaffolds (**A**) and within the geometrically spaced pores (**B** and **C**). The scale bar is 100 μm

The viability of cells on patterned PEGDA hydrogels was assessed by culturing ATDC5 cells on collagen-coated PEGDA microscaffolds (Fig. 7A), and in the porous spaces within the microscaffolds following delivery with a collagen hydrogel for 14 days (Fig. 7B and C). Cells in collagen responded well to the biomaterial, engrafting to the collagen-coated surface and growing within the pores to form a connected network of cells after 14 days, and validating our overall approach using self-assembling 4D printing as a tissue engineering strategy for supporting cartilage defect repair. After 7 days culture, cells seeded directly onto uncoated, native PEGDA were viable but aggregated (supplementary fig. S1 A & B), corresponding to other published studies that pure PEGDA is bio-inert, i.e. is not cytotoxic but provides no chemically suitable surface for cells to attach [51]. Interestingly, a 7-day experiment in which chondrocytes were cultured on PEGDA thin sheet scaffolds with nanocellulose fibres appeared to show an increase in cell attachment, spreading and growth, indicating a potential role for enhanced bioactivity caused by nanocellulose fibres admixed into PEGDA, although it was not determined if this effect was due to biochemical or nano-topographical improvements to the surface properties of the material (supplementary fig. S1 C & D).

The cytotoxicity of the photoinitiator LAP was assessed by the LDH assay, which commonly detects cell death by measuring the release of cytoplasmic LDH enzyme from dead cells (Supplementary fig. S2). Three cell lines (fibroblasts, myoblasts and chondrocytes) displayed different levels of sensitivity to LAP, with chondrocytes proving the most sensitive, particularly to the highest concentration of LAP. Both myoblasts and fibroblasts were relatively more tolerant, and fibroblasts viability minimally impacted even by the higher concentrations, and without the dose-dependent sensitivity observed most clearly in myoblasts. From these results, 0.05% LAP displayed the lowest level of cytotoxicity on the viability and proliferation of all three cell lines in vitro, which was selected in the formula of printing ink to fabricate the patterned microscaffolds.

Previous studies have demonstrated that small molecular weight residues following photo-crosslinking of PEGDA can cause cytotoxicity if not removed between fabrication and cell culture. Commonly known as leaching, the process can be expedited by immersion in aqueous solutions to actively remove these cytotoxic species prior to cell culture. To investigate the level of toxicity caused by leaching (and to determine the number of wash steps required post-fabrication), we assessed a series of wash solutions after incubating freshly manufactured PEGDA microscaffolds and quantified cell viability in these solutions by biomaterials ISO standard MTT assay. Most experimental groups showed less than 70% viability in the first wash solution (leachates from the first 72 h post-fabrication), indicating that these initial leaching species have a high level of toxicity to cells. However, the second conditioned medium (containing leachate from 72 to 144 h washing) presented a greatly reduced toxic effect on the viability of cells in vitro (supplementary fig. S2). These data are consistent with previous studies [52, 53], suggested that PEGDA released cytotoxic species after DLP-3D printing and photopolymerisation, but these can be easily removed by a series of aqueous wash steps. In this and future studies, 3D-printed microscaffolds were all stored in pure water for 3 days after printing, prior to biological experiments.

## Conclusions

Patterned PEGDA microscaffolds were successfully fabricated using a customised DLP-3D printer in a range of sizes and geometries. Hexagonal microscaffolds were shown to have self-assembly properties resulting from effective tessellation without significant overlap piping when delivered in an aqueous colloidal suspension with appropriate rheological properties. Microscaffolds 0.75 mm in diameter and 0.3 mm in thickness with a hexagonal contour profile showed the most convincing self-assembly properties when suspended in a 1% methyl cellulose (~ 7.5cp) solution, covering 73.3% of a 1 cm diameter simulated chondral lesion. We determined that this auto-tessellation property fulfils the requirements of a 4D printing methodology, in which a 3D-printed microscaffold can spontaneously organise into a macrostructural superscaffold with requisite properties, in this case to provide a mechanically stable defect repair with a compressive modulus matching the native tissue, plus a sufficiently porous structure to support cellular engraftment prior to tissue regeneration. Freshly fabricated PEGDA microscaffolds were shown to be slightly cytotoxic, but serial wash steps were sufficient to remove any residual toxicity. We demonstrated that cells can be successfully engrafted into porous PEGDA microscaffolds and survive for at least 14 days with no signs that future cell growth will be inhibited. This study demonstrates the feasibility of miniaturised micro-structured hydrogel scaffold to self-assemble into a macrostructural cell-compatible biomaterial. With further development, this presents a promising injectable strategy for stratified in situ reconstruction of cartilage lesions using a simple arthroscopic procedure.

### Supplementary Information

Below is the link to the electronic supplementary material.

Supplementary file1 (PPTX 1778 kb)

## Data Availability

The datasets generated during and/or analysed during the current study are available from the corresponding author on reasonable request.
