# Supplementary material for: A 4D printed self-assembling PEGDA microscaffold fabricated by digital light processing for arthroscopic articular cartilage tissue engineering
Source: Prog Addit Manuf. 2022 Nov 9;9(1):3–14. doi: 10.1007/s40964-022-00360-0 (PMC10851926; doi:10.1007/s40964-022-00360-0)
Supplement: Supplementary file 1 — Supplementary file1 (PPTX 1778 kb) [file 40964_2022_360_MOESM1_ESM.pptx]

## Slide 1
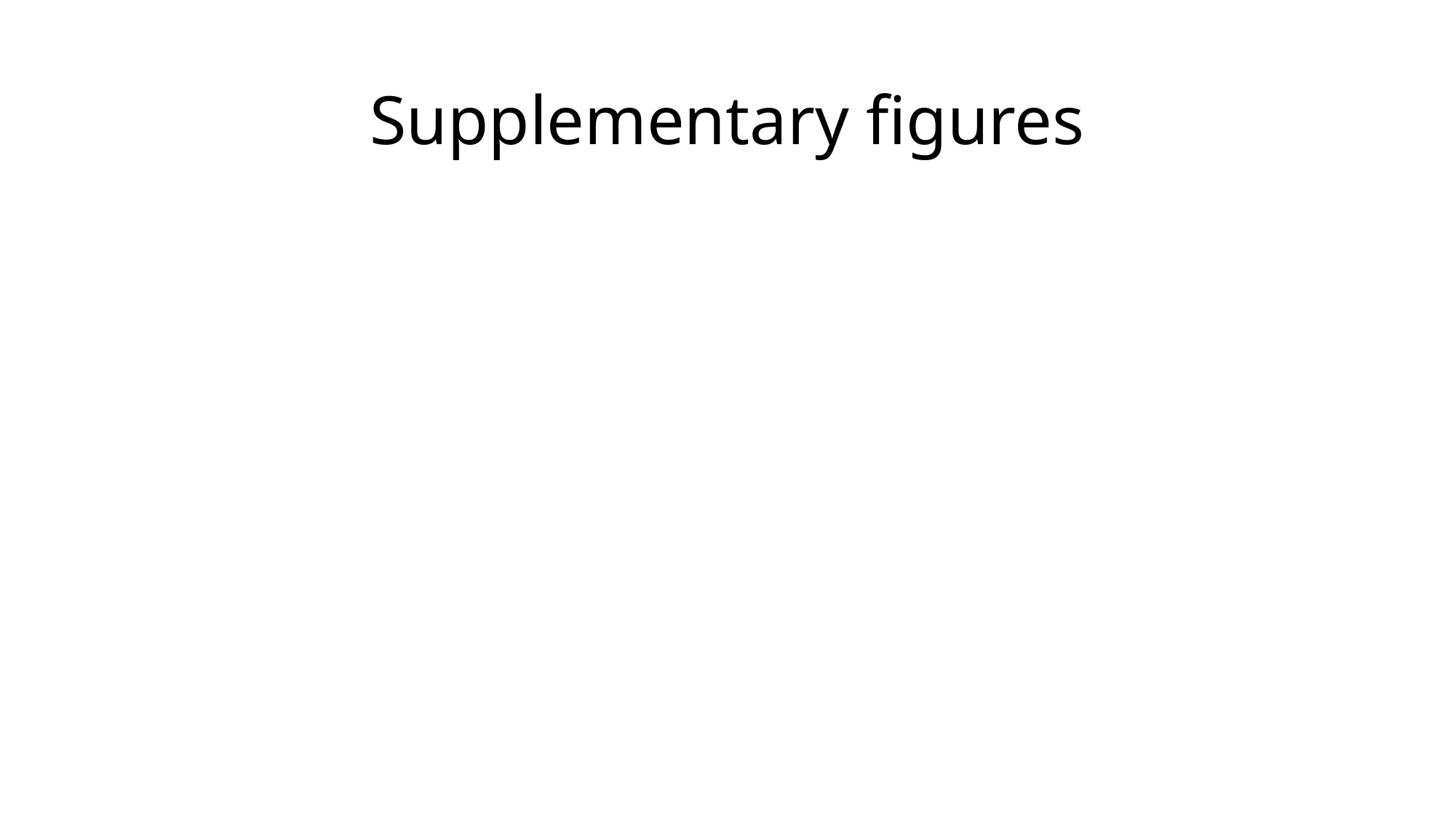

# Supplementary figures

## Slide 2
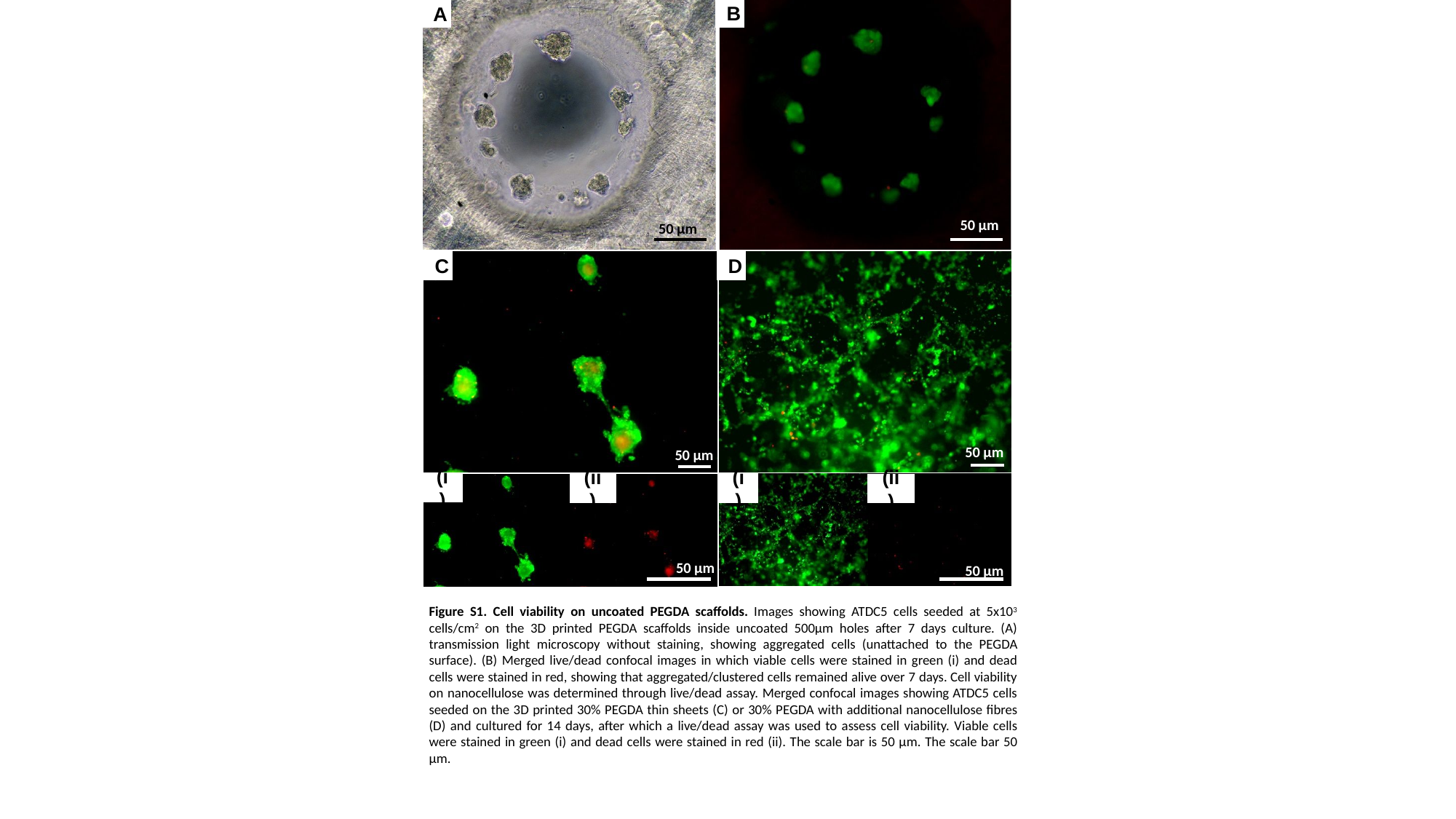

B
A
50 µm
50 µm
D
C
50 µm
50 µm
(i)
(ii)
(i)
(ii)
50 µm
50 µm
Figure S1. Cell viability on uncoated PEGDA scaffolds. Images showing ATDC5 cells seeded at 5x10^3^ cells/cm2 on the 3D printed PEGDA scaffolds inside uncoated 500μm holes after 7 days culture. (A) transmission light microscopy without staining, showing aggregated cells (unattached to the PEGDA surface). (B) Merged live/dead confocal images in which viable cells were stained in green (i) and dead cells were stained in red, showing that aggregated/clustered cells remained alive over 7 days. Cell viability on nanocellulose was determined through live/dead assay. Merged confocal images showing ATDC5 cells seeded on the 3D printed 30% PEGDA thin sheets (C) or 30% PEGDA with additional nanocellulose fibres (D) and cultured for 14 days, after which a live/dead assay was used to assess cell viability. Viable cells were stained in green (i) and dead cells were stained in red (ii). The scale bar is 50 µm. The scale bar 50 µm.

## Slide 3
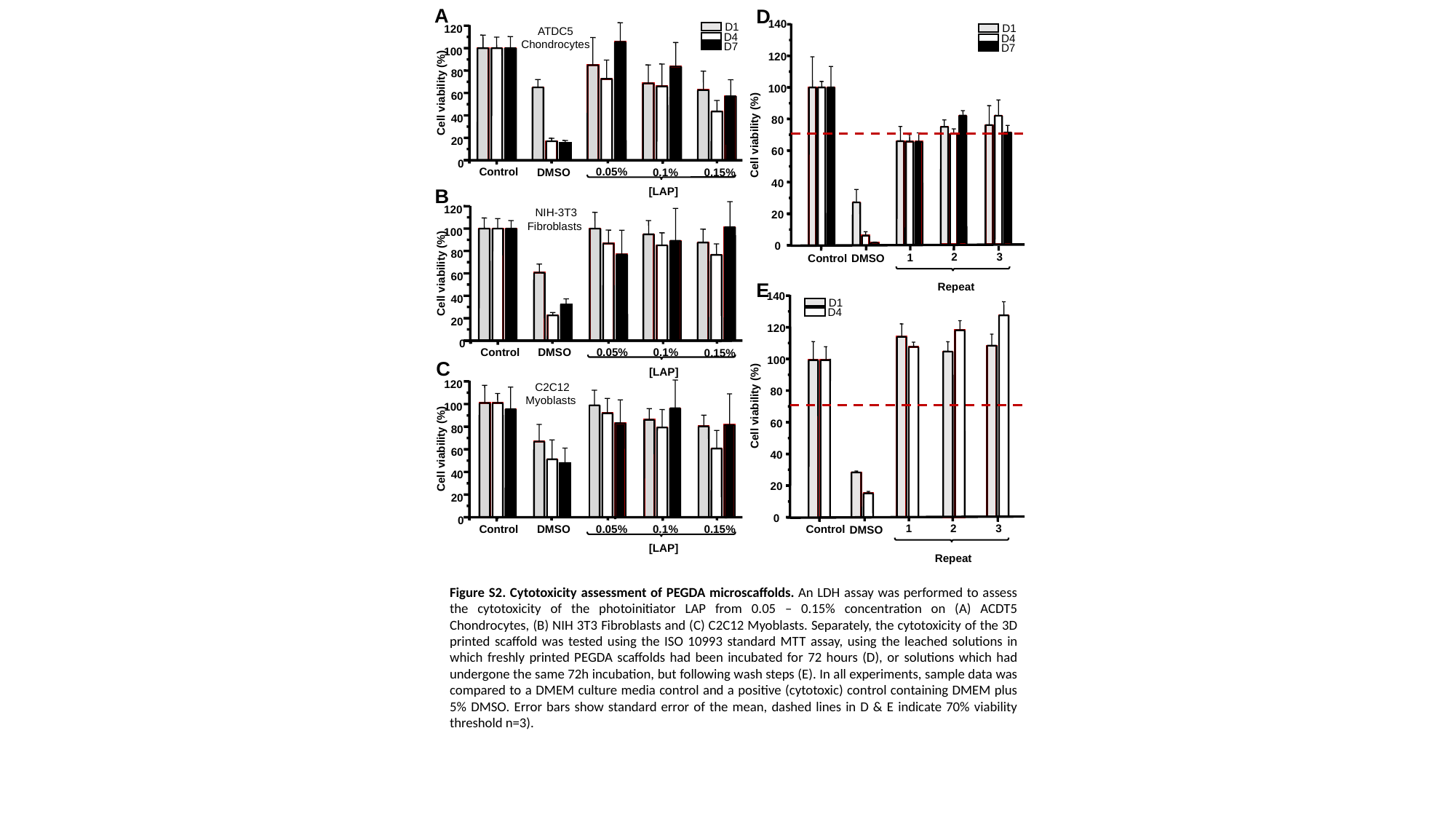

A
D
140
120
100
80
60
40
20
0
Cell viability (%)
2
3
1
Control
DMSO
Repeat
 D1
D4
D7
120
 ATDC5
Chondrocytes
100
80
Cell viability (%)
60
40
20
0
Control
0.05%
DMSO
0.1%
0.15%
[LAP]
 D1
D4
D7
B
120
100
80
Cell viability (%)
60
40
20
0
Control
0.05%
DMSO
0.1%
0.15%
[LAP]
 NIH-3T3
Fibroblasts
E
140
 D1
D4
120
100
80
60
40
20
0
2
3
1
Control
DMSO
Repeat
Cell viability (%)
C
120
100
80
Cell viability (%)
60
40
20
0
Control
0.05%
DMSO
0.1%
0.15%
[LAP]
 C2C12
Myoblasts
Figure S2. Cytotoxicity assessment of PEGDA microscaffolds. An LDH assay was performed to assess the cytotoxicity of the photoinitiator LAP from 0.05 – 0.15% concentration on (A) ACDT5 Chondrocytes, (B) NIH 3T3 Fibroblasts and (C) C2C12 Myoblasts. Separately, the cytotoxicity of the 3D printed scaffold was tested using the ISO 10993 standard MTT assay, using the leached solutions in which freshly printed PEGDA scaffolds had been incubated for 72 hours (D), or solutions which had undergone the same 72h incubation, but following wash steps (E). In all experiments, sample data was compared to a DMEM culture media control and a positive (cytotoxic) control containing DMEM plus 5% DMSO. Error bars show standard error of the mean, dashed lines in D & E indicate 70% viability threshold n=3).

## Slide 4
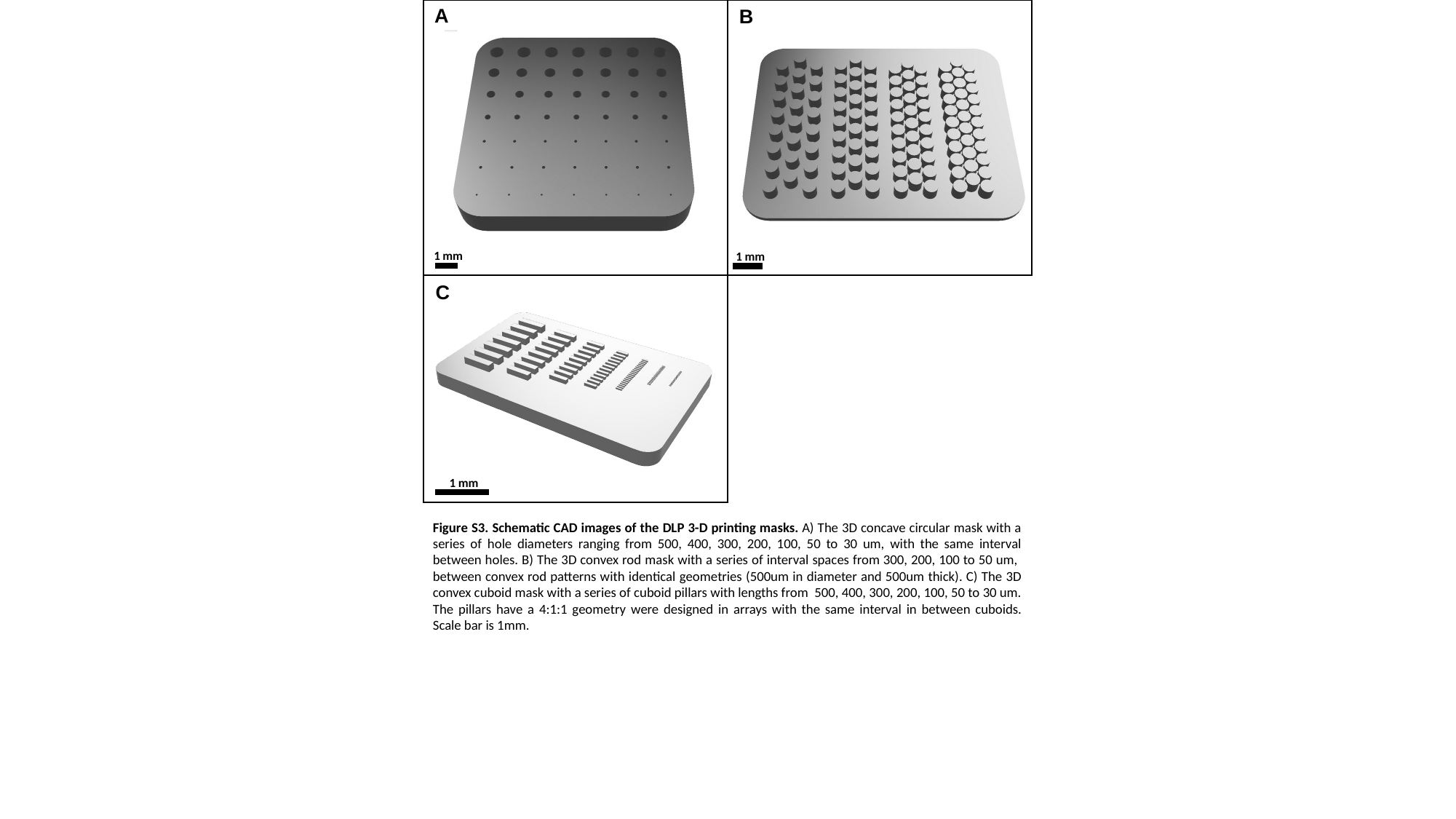

A
B
1 mm
1 mm
C
1 mm
Figure S3. Schematic CAD images of the DLP 3-D printing masks. A) The 3D concave circular mask with a series of hole diameters ranging from 500, 400, 300, 200, 100, 50 to 30 um, with the same interval between holes. B) The 3D convex rod mask with a series of interval spaces from 300, 200, 100 to 50 um, between convex rod patterns with identical geometries (500um in diameter and 500um thick). C) The 3D convex cuboid mask with a series of cuboid pillars with lengths from 500, 400, 300, 200, 100, 50 to 30 um. The pillars have a 4:1:1 geometry were designed in arrays with the same interval in between cuboids. Scale bar is 1mm.
